# How could mouthwashes affect the color stability and translucency of various types of monolithic zirconia? An in-vitro study

**DOI:** 10.1371/journal.pone.0295420

**Published:** 2023-12-01

**Authors:** Rashin Giti, Reza Jebal

**Affiliations:** 1 Department of Prosthodontics, Faculty of Dentistry, Shiraz University of Medical Sciences, Shiraz, Fars, Iran; 2 Student Research Committee, Faculty of Dentistry, Shiraz University of Medical Sciences, Shiraz, Fars, Iran; Yerevan State Medical University Named after Mkhitar Heratsi, ARMENIA

## Abstract

**Objectives:**

This study aimed to evaluate the color stability and translucency of various types of monolithic zirconia after immersion in chlorhexidine and Listerine mouthwashes.

**Materials and methods:**

This experimental study was performed on 36 disk-shaped specimens fabricated from low-translucent, high-translucent, and multilayered monolithic zirconia (*n* = 12 per group). Each group was equally divided and immersed in either 2% chlorhexidine (CHX) or Listerine mouthwash for 2 min daily over 7 days. Changes in color (ΔE) and the translucency parameter (ΔTP) were evaluated and compared. The data were analyzed with one-way ANOVA followed by Tukey’s post-hoc tests (*α* = 0.05).

**Results:**

Chlorhexidine caused a significantly lower ΔE and a significantly higher ΔTP in multilayered zirconia compared to the low-translucency (ΔE: *P* = 0.0027, ΔTP: *P*<0.001) and the high-translucency zirconia group (ΔE: *P*<0.001, ΔTP: P = 0.022). Listerine caused a significantly higher ΔE in the high-translucency zirconia group compared to the multilayered zirconia group (*P* = 0.0165). It also resulted in a significantly higher mean ΔTP in the multilayered zirconia group compared to the low-translucency (*P* = 0.0003) and high-translucency zirconia groups (*P* = 0.019).

**Conclusions:**

In both mouthwashes, multilayered monolithic zirconia exhibited the highest color stability among the tested materials; albeit with the most pronounced changes in translucency. Meanwhile, high-translucency monolithic zirconia was more prone to discoloration when exposed to both mouthwashes.

## 1. Introduction

When prioritizing aesthetics, all-ceramic restorations emerge as the optimal material of choice, due to their ability to simulate the optical characteristics of natural teeth [1, 2]. Among all-ceramic prosthetic materials, zirconium oxide (ZrO_2_), serving as the base material, stands out for its excellent mechanical properties and the unique transformation toughening phenomenon [3, 4]. All-ceramic zirconia restorations are mainly categorized as bilayered core-ceramic and monolithic zirconia. Notably, monolithic yttrium-stabilized tetragonal zirconia (Y-TZP) crowns exhibit higher fracture resistance than bilayered veneered Y-TZP crowns [5, 6]. Monolithic zirconia restorations offer distinct benefits, including a streamlined fabrication process that enhances cost and time efficiency, as well as obviating the need for a veneering layer, which effectively prevents chipping. However, a notable drawback lies in their lower esthetic properties compared to other ceramic systems [7, 8].

Monolithic zirconia can be esthetically enhanced by increasing translucency through elevating yttria stabilizer levels [9], reducing grain size and sintering duration [10], and incorporating 0.2 mol% La_2_O_3_ into the composition [11, 12]. Higher yttria contents results in a greater proportion of crystals maintaining the cubic phase post-cooling, thereby enhancing translucency. Additionally, a higher cubic phase content reduces the tetragonal-to-monoclinic phase transition, leading to reduced low-temperature degradation. However, modifying the composition to improve translucency may diminish the material’s flexural strength and fracture toughening properties [13, 14]. Multi-layered monolithic zirconia mimics the natural shade-gradient of teeth, which is achieved by either pigmenting zirconia within each blank from the same generation, or using a zirconia of high flexural strength in the body/dentin area and one of a higher translucency in the incisal/occlusal area [15–17].

Despite their increasing popularity, tooth-colored restorations are susceptible to discoloration due to the consumption of items like colored beverages or mouthwashes. Meanwhile, mouthwashes are essential for chemical plaque control as an adjunct to mechanical plaque control, particularly in individuals with high-risk caries or susceptibility to periodontal diseases [18–23]. Notwithstanding the proven antiseptic efficacy of chlorhexidine mouthwash, it is associated with side effects such as taste impairment and staining of teeth, mucosa, and restorative materials [24–28]. In attempts to investigate the antibacterial effects of mouthwashes containing essential oils, like Listerine, notable efficiency has been observed in diminishing plaque and gingivitis, complementing mechanical plaque control [29–32]. Nonetheless, the presence of alcohol and low pH in Listerine can lead to enamel discoloration and demineralization [33–35].

Presently, discoloration stands out as a leading clinical reason (constituting 38% of cases) for prosthesis replacement. Discoloration can be assessed through visual examination or digital instruments. A color change exceeding 3.7is deemed clinically unacceptable and may require restoration replacement, as it is perceptible to normal eyes [7].

The visual assessment of color is inherently subjective due to physiological and psychological factors, including object/observer’s position relative to illumination and the observer’s emotional state. This subjectivity and the associated errors can be eliminated by using spectrophotometer, which measures color through reading all three color components (L*, a*, b*), regardless of the surface type [34]. Translucency is defined by the scattering of light wavelength. If most wavelengths of light are scattered, a ceramic looks opaque; if most are transmitted, it appears translucent [36].

Derafshi et al. [20] reported that the immersion of monolithic zirconia and feldspathic ceramic in 0.2% chlorhexidine digluconate and Listerine (2 min daily for 7 days) caused discoloration in both restorative materials, compared to immersion in distilled water. Sasany et al. [37] found significant discoloration in two 5Y-TZP zirconia types immersed in chlorhexidine and Listerine. While discoloration was clinically acceptable in crown thickness for both zirconia materials, Zirkonzahn high-translucent zirconia showed clinically unacceptable discoloration in veneer thickness when exposed to Listerine. Moreover, translucency reduced in laminate veneer thickness for both zirconia materials after immersion in Listerine. Another study showed that one week of immersion in an acidic drink did not cause perceptible discoloration in CAD-CAM zirconia ceramics [38].

Considering the limited research and controversies surrounding changes in the translucency parameter of monolithic zirconia after immersion in mouthwashes, and the lack of studies on the effect of different types of mouthwashes on the color stability of various monolithic zirconia systems with differing translucencies, this study sought to evaluate the impact of chlorhexidine and Listerine mouthwashes on the color stability and translucency of different monolithic zirconia systems. The null hypothesis posited that mouthwashes would not affect the color stability and translucency of different monolithic zirconia systems.

## 2. Materials and methods

### 2.1. Fabrication of specimens

In this experimental *in-vitro* study, 36 disk-shaped specimens with dimensions of 2×15 mm (thickness×diameter) were designed (CAD design software; 3shape, Copenhagen, Denmark) and milled (CAD-CAM machine, CORiTEC 340i; imes-icore GmbH, Eiterfeld, Germany) out of three types of pre-sintered monolithic zirconia (n = 12 per group) including low-translucency monolithic zirconia (LT) (DD Bio ZW iso, High Strength Zirconia, Dental Direkt, Germany), high-translucency monolithic zirconia [8] (DD Bio ZX^2^ 98, High Translucent Zirconia, Dental Direkt, Germany), and multilayered monolithic zirconia (ML) (DD cubeX^2^^®^ML, Multilayer, Cubic Zirconia System, Dental Direkt, Germany).

**Fig 1:**
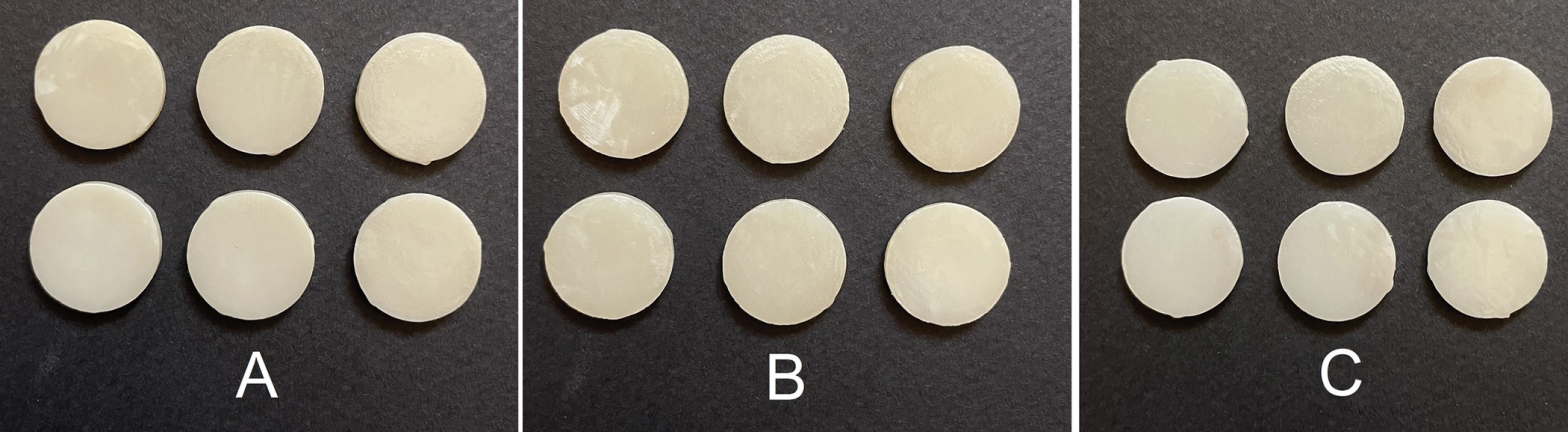
Monolithic zirconia samples of each subgroup before immersion in mouthwashes: A) low-translucency monolithic zirconia, B) high-translucency monolithic zirconia, C) multilayered monolithic zirconia.

The specimens were manufactured according to ISO 6872, with an accuracy of ± 0.02 mm [39]. Multilayered specimens were pre-colored by the manufacturer, while high-translucency and low-translucency zirconia specimens were shaded with coloring liquids in the laboratory. To standardize the initial color for all specimens, shade A2 was chosen from the VITA shade guide (Vita ZahnFabrik, Bad Säckingen, Waldshut, Germany) (Fig 1). Subsequently, the specimens were sintered following the manufacturer’s instructions for 8 h at 1450°C. After sintering, both surfaces of the specimens were uniformly polished according to the manufacturer’s instructions. Then, the specimens were ultrasonically cleaned in distilled water for 15 min before testing and individually air-dried for 30 s.

### 2.2. Color measurement

Baseline color values (L*, a*, b*) were measured by using a reflectance spectrophotometer (VITA Easyshade V^®^, Bad Säckingen, Waldshut, Germany), which was calibrated before each measurement according to the manufacturer’s instructions. The spectrophotometer’s CIELab output was based on D65 illuminant and a 2-degree standard observer. Three measurements were taken for each specimen, and the mean value was calculated. The color and translucency measurements were performed at the same time of day by a single technician who was blinded to the study groups.

The color measurements were taken against a neutral gray background, while the translucency measurements were conducted against black (b) and white (w) backgrounds. To ensure consistent conditions, the device was positioned perpendicular to each specimen. The Commission International de l’Éclairage introduced a color reading system, where L* represents the luminosity axis, a* represents the green-red axis (-a = green, +a = red), and b* represents the blue-yellow axis (-b = blue, +b = yellow). Translucency was measured against black (b) and white (w) backgrounds, with the translucency parameter being calculated using the following formula [40]:

Translucency parameter (TP) = ([L_b_−L_w_]^2^ + [a_b_−a_w_]^2^ + [b_b_−b_w_]^2^)^½^

Each group of monolithic zirconia systems (HT, LT, and ML) was equally divided into subgroups (n = 6) to be immersed in either 10 ml of 0.2% chlorhexidine mouthwash (G1) (Vi-one^®^, Rojin Cosmetic Co., Tabriz, Iran) or 10 ml of Listerine mouthwash (G2) (Listerine^®^, Intense Freshness, Johnson & Johnson, Neuss, Germany) for 2 min daily over 7 days [20]. According to a previous study [20] and considering a 5% significance level and power (1-β) of 80%, and sd1 = 0.2, sd2 = 0.12, mean difference (d) = 0.33 and one by one ratio (r) = 1 with the formula of: n=1+rrs2(z1−α2+z1−β)2(d)2, the sample size was estimated 6 per each subgroups (12 in each of 3 groups). Hence, a total of 36 samples was needed per the following formula: $s^{2}\approx \frac{sd_{1}^{2}+{sd}_{2}^{2}}{2}$.

The specimens were stored in separate containers filled with physiological saline solution between daily mouthwash exposures; and both the mouthwash and physiological solutions were renewed daily. The study groups were as follows: G1HT (high-translucent monolithic zirconia in chlorhexidine mouthwash), G1LT (low-translucent monolithic zirconia in chlorhexidine mouthwash), G1ML (multilayered monolithic zirconia in chlorhexidine mouthwash), G2HT (high-translucent monolithic zirconia in Listerine mouthwash), G2LT (low-translucent monolithic zirconia in Listerine mouthwash), and G2ML (multilayered monolithic zirconia in Listerine mouthwash).

After 7 days, color and translucency were remeasured as previously described. Each specimen was then washed with distilled water and dried with paper. The total color change (ΔE_ab_) was calculated using the following formula [34]:

ΔE = [(ΔL)^2^ + (Δa)^2^ + (Δb)^2^]^1/2^

ΔTP was calculated as follows: (TP after immersion -TP before immersion)

Color difference is considered imperceptible when 0.5<ΔE<1, and the threshold for a perceptible color difference is ΔE = 1. A ΔE≥3.7 is the value for a color difference visible to the naked eye, rendering ΔE = 3.7 the established clinically-acceptable threshold for color change [40].

### 2.3. Statistical analysis

The data were analyzed by using SPSS software (Version 16.0; SPSS Inc., Chicago, United States). The results were presented as mean ± standard error (SE) for ΔE and ΔTP. The Shapiro-Wilk test was used to evaluate normal distribution, and Levene’s test was used to assess the equality of variance. One-way ANOVA with Tukey’s post hoc was used to compare the groups (α = 0.05).

## 3. Results

The Shapiro-Wilk test approved the normal distribution and Levene’s tests confirmed the equality of variances (Table 1).

**Table 1:** P values extracted from Shapiro-Wilk and Levene’s tests for checking the normality and equality of variances.

P values Tested factors		Normality			Equality of variances
		Low-translucency zirconia	High-translucency zirconia	Multilayered zirconia	
ΔE	Chlorhexidine	0.804	0.641	0.819	0.686
	Listerine	0.578	0.695	0.289	0.916
ΔTP	Chlorhexidine	0.356	0.518	0.417	0.620
	Listerine	0.283	0.869	0.201	0.714

### 3.1. Chlorhexidine

**Fig 2:**
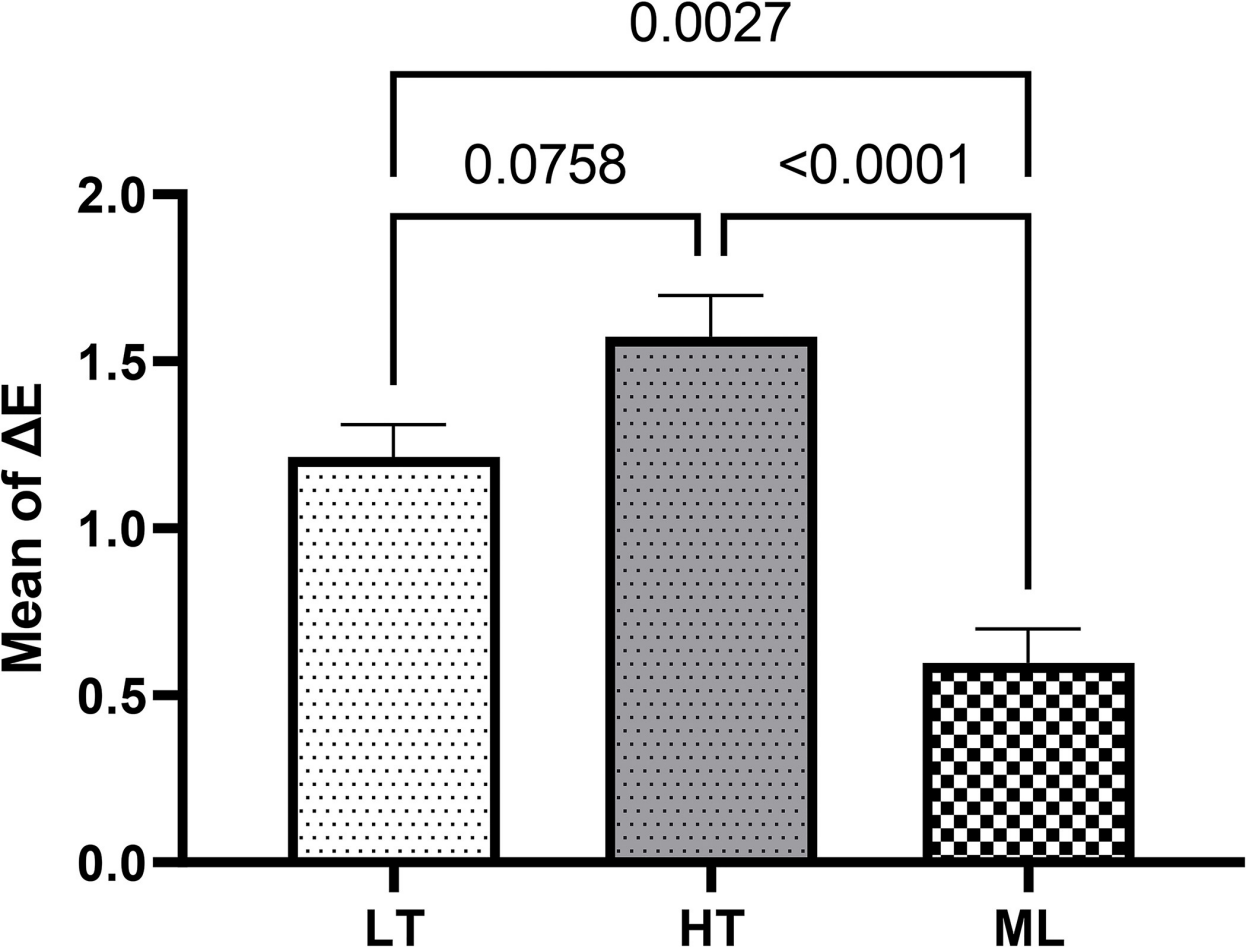
Comparing the mean (±standard error) of ΔE caused by chlorhexidine mouthwash among the study groups.

**Fig 3:**
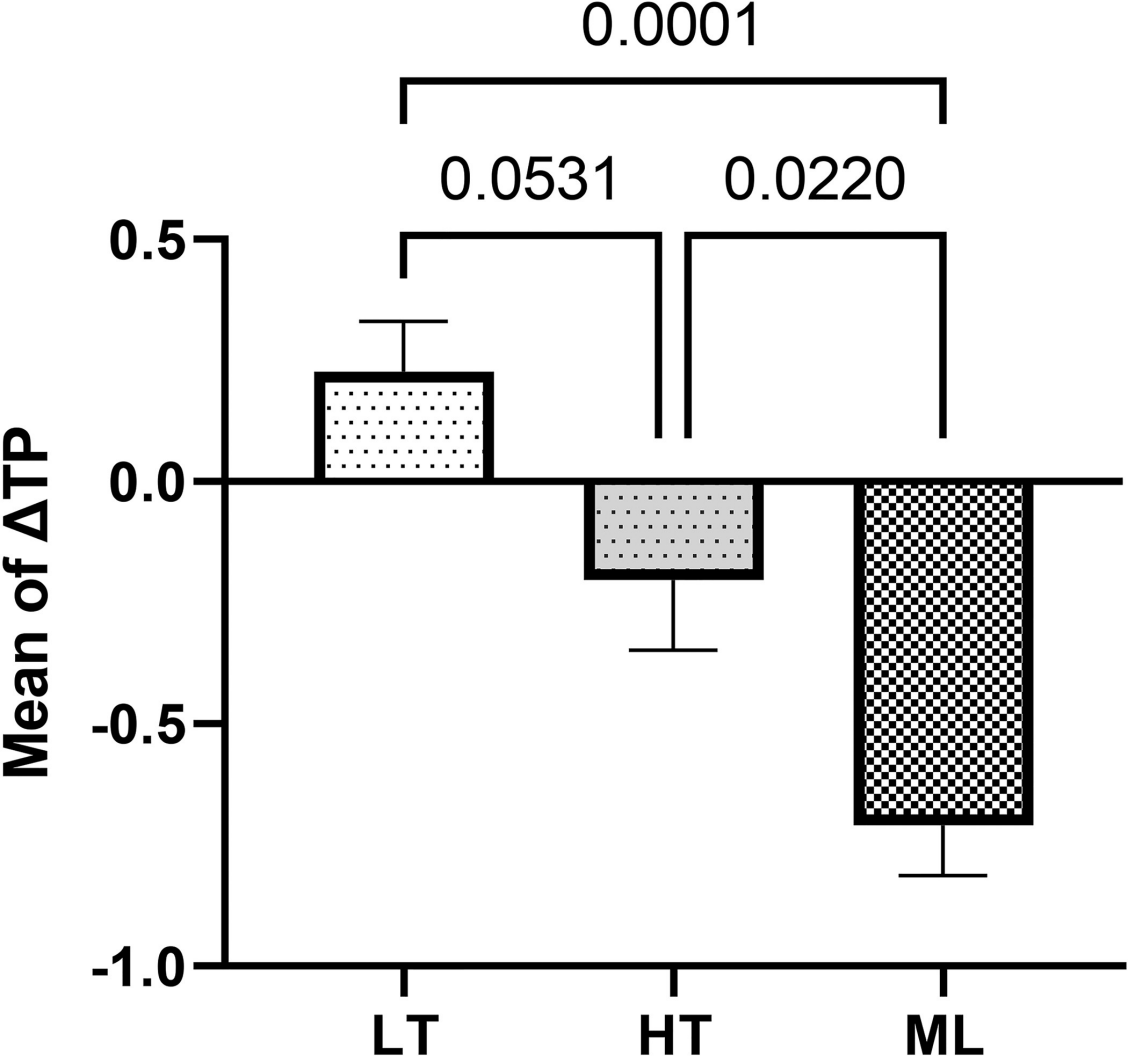
Comparing the mean (±standard error) of ΔTP caused by chlorhexidine mouthwash among the study groups.

The ANOVA results revealed that the three zirconia groups immersed in chlorhexidine were significantly different in terms of ΔE (F = 21.2, *P*<0.001) and ΔTP (F = 15.6, *P*<0.001). The results of Tukey’s post hoc test showed that ΔE of the ML zirconia was significantly lower than that of the LT zirconia (*P* = 0.0027) and HT zirconia groups (*P*<0.001). The mean ΔTP of ML zirconia was significantly different from that of the LT zirconia (*P*<0.001) and HT zirconia (*P* = 0.022). Chlorhexidine mouthwash increased the translucency parameter in the LT zirconia and decreased it in the HT zirconia and ML zirconia groups (Table 2, Figs 2 and 3).

**Table 2:** Mean and standard error of ΔE and ΔTP cause by chlorhexidine mouthwash.

Chlorhexidine	Groups			F	P value
	Low-translucency zirconia	High-translucency zirconia	Multilayered zirconia		
ΔE	1.21 ± 0.09	1.57 ± 0.12	0.59 ± 0.10	21.2	<0.001
ΔTP	0.22 ± 0.10	-0.20 ± 0.14	-0.71 ± 0.10	15.6	<0.001

### 3.2. Listerine

**Fig 4:**
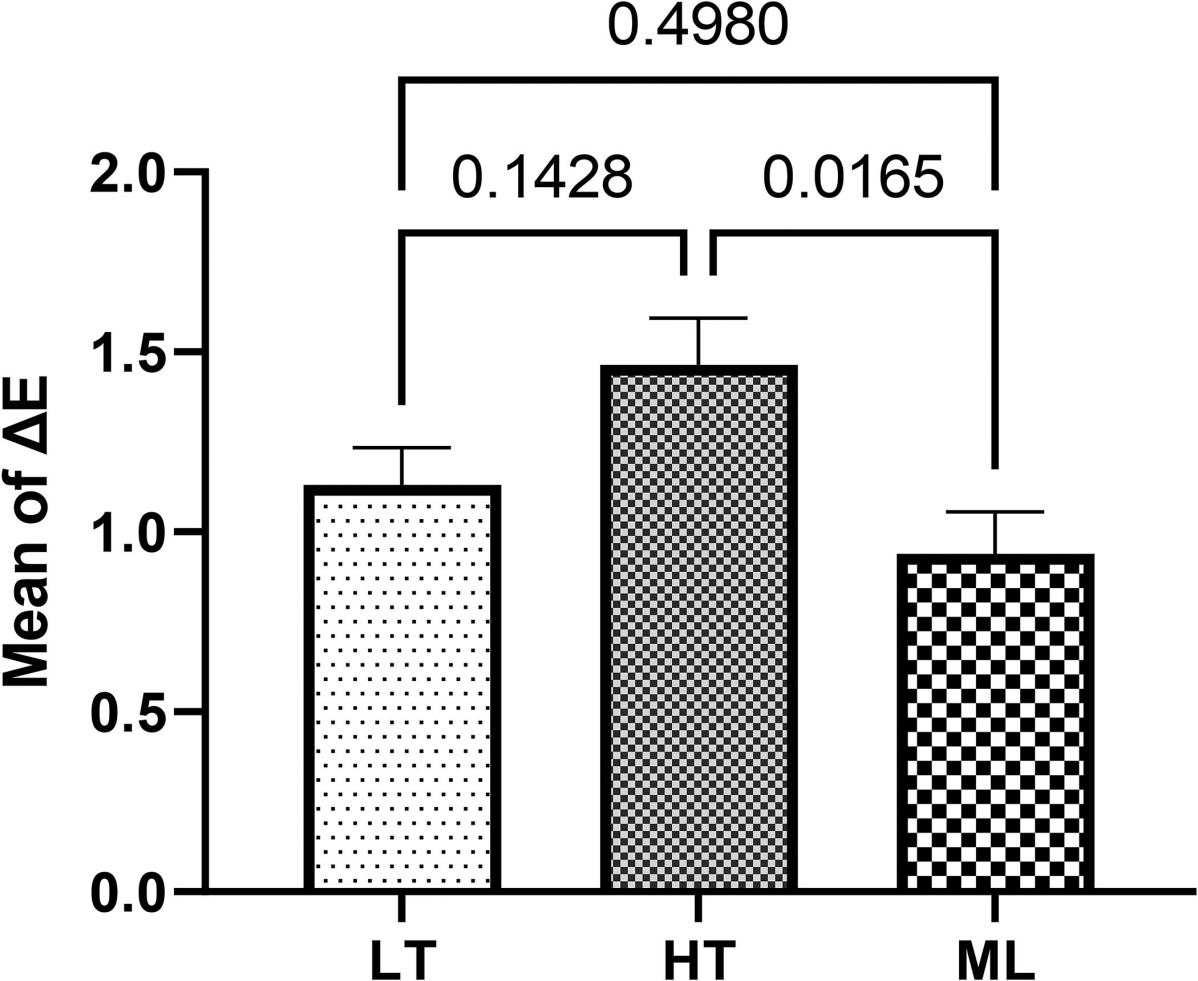
Comparing the mean (±standard error) of ΔE caused by Listerine mouthwash among the study groups.

**Fig 5:**
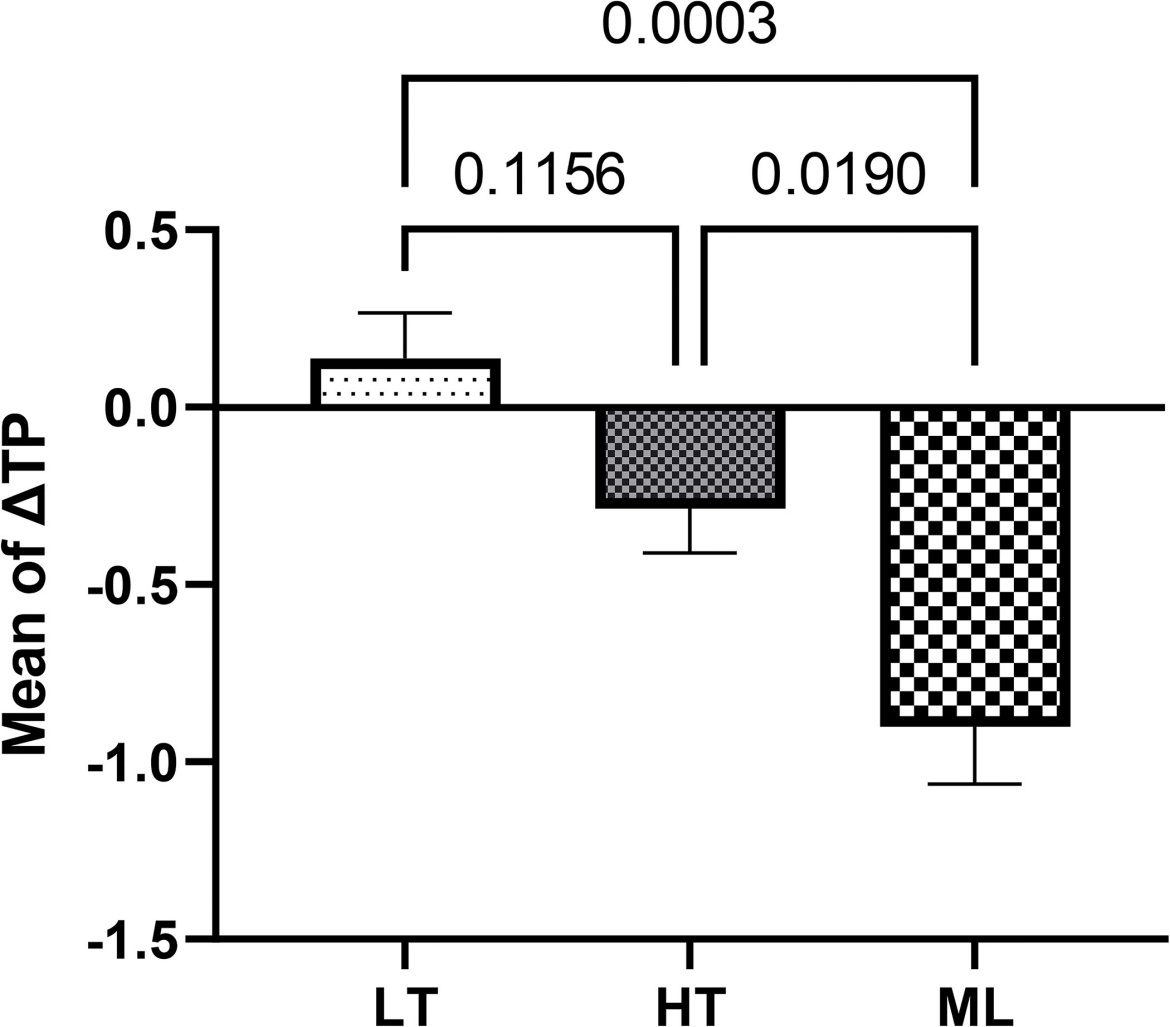
Comparing the mean (±standard error) of ΔTP caused by Listerine mouthwash among the study groups.

According to the ANOVA results, Listerine mouthwash caused the three groups to be significantly different in terms of ΔE (F = 5.1, *P* = 0.020) and ΔTP (F = 13.8, *P*<0.001). The results of Tukey’s post hoc test showed that ΔE of the HT zirconia was significantly higher than that of the ML zirconia (*P* = 0.0165). Moreover, the mean ΔTP of ML zirconia was significantly different from that of the LT (*P* = 0.0003) and HT zirconia groups (*P* = 0.019). Listerine mouthwash increased the translucency parameter in the LT group and decreased it in the HT and ML zirconia groups (Table 3, Figs 4 and 5).

**Table 3:** Mean and standard error of ΔE and ΔTP caused by Listerine mouthwash.

Listerine	Groups			F	P value
	Low-translucency zirconia	High-translucency zirconia	Multilayered zirconia		
ΔE	1.13 ± 0.10	1.46 ± 0.12	0.93 ± 0.11	5.1	0.020
ΔTP	0.13 ± 0.12	-0.28 ± 0.12	-0.90 ± 0.16	13.8	<0.001

## 4. Discussion

The null hypothesis was rejected as both mouthwashes affected the color stability and translucency of different types of monolithic zirconia. Irrespective of the mouthwashes, the highest and lowest color changes were seen in the high-translucency and multilayered zirconia groups, respectively. Nevertheless, color changes in all groups remained within the clinically acceptable range (ΔE< 3.7) [40]. In terms of ΔTP, both chlorhexidine and Listerine prompted the most substantial changes in the translucency parameter for multilayered zirconia. In chlorhexidine mouthwash, ΔTP was the lowest in high-translucency zirconia; while in Listerine mouthwash, ΔTP was the lowest in low-translucency zirconia group. Both mouthwashes increased the translucency parameter in the low-translucency zirconia and decreased it in the multilayered and high-translucency groups. However, the high-translucency and low-translucency zirconia groups did not show statistically significant differences in terms of ΔE and ΔTP.

Amid concerns regarding color and translucency in monolithic zirconia, several studies have assessed the influence of mouthwashes on these aspects [20, 37, 41]. Consistent with the current study, Derafshi et al. [20] found that while immersing monolithic zirconia and feldspathic ceramic in distilled water did not significantly affect the color, chlorhexidine and Listerine had significant impacts; although color changes remained below the clinically acceptable threshold. Similarly, in line with the present study, Alnassar [42] observed that among the studied staining liquids, 28 days of exposure to coffee caused the highest discoloration in high-translucency monolithic zirconia. Marked color change in chlorhexidine was noted after 14 days; however, it remained within the clinically acceptable range. The higher discoloration, in comparison with the current study, might be due to the longer exposure time.

Sasany et al. [37] detected that both 5Y-TZP zirconia and lithium disilicate specimens with 0.7-mm-thick laminate veneer experienced significant color changes in both Klorhex and Listerine; although almost all color changes were esthetically clinically acceptable. Consistent with the present findings for multilayered and high-translucency zirconia, their findings demonstrated that 0.7-mm-thick specimens experienced significant decreases in translucency in both zirconia groups when exposed to Listerine. However, no significant translucency change occurred in 1.5-mm-thick specimens. This suggests a potential correlation between the zirconia specimens’ thickness and translucency alterations.

The variations in ΔE and ΔTP among different monolithic zirconia materials in the current study may stem from factors such as variations in chemical structures, grain size and shape, crystalline phase distribution, porosity, and thickness, all of which can affect zirconia’s optical properties [43–46]. The present study employed materials of the same thickness. In higher translucent zirconia types, manufacturers either decrease alumina content or increase the yttria stabilizer amount, which, in turn, leads to greater proportion of crystals retaining the cubic phase after cooling [13, 47].

Multilayered zirconia mimics the shade gradient of natural teeth, with the incisal area of a crown displaying the highest translucency and gradually increasing in opacity and chroma towards the gingival area [48]. The layered structure of multilayered zirconia is related to different material properties of the individual layers [49]. In the present study, low-translucency specimens were 3Y-TZP, and high-translucency specimens were 3Y-TZP with added La_2_O_3_ dopants, whereas multilayered specimens were 5Y-TZP. Adding La_2_O_3_ to conventional zirconia increases translucency by promoting smaller grain sizes and narrowing grain boundaries. However, in such materials, the reduction of alumina content is essential to preserve mechanical properties. This trade-off can decrease aging stability, as evidenced by the high-translucency zirconia group in the present study [12, 50, 51].

Consistent with the lowest color change observed in multilayered zirconia specimens in the current study, similar research showed that higher 5Y-TZP cubic zirconia yielded lower ΔE compared to 3Y-TZP tetragonal specimens. Existing literature suggests that elevated yttria content can minimize surface-level low-temperature degradation, potentially reducing surface roughness and solution infiltration [8, 11, 52].

In the present study, the multilayered zirconia specimens were pre-colored by the manufacturer, while the low-translucency and high-translucency zirconia specimens were shaded with coloring liquids in the laboratory. In line with the current findings indicating the lowest ΔE in multilayered zirconia specimens, Subaşı et al.’s findings [53] demonstrated that coffee could cause less color changes in thermocycled pre-shaded monolithic and veneered zirconia than in the externally-shaded zirconia specimens. They also found that the transparency parameter was affected by the shading technique, potentially contributing to the greatest ΔTP in multilayered zirconia specimens in the present study. Another possibility is that in multilayered zirconia, the mismatch in thermal expansion coefficient between layers could generate residual stresses at their interfaces during post-sintering cooling [54]. It is speculated that mouthwashes could render the layers interface more susceptible to microstructural changes and pore formation, causing light scattering and consequently decreasing the translucency parameter.

Unlike the present study, Lee et al. [41] reported that 180 hours of exposure to chlorhexidine and Listerine mouthwashes had no significant effect on the mean translucency parameter of high-translucency zirconia. This contrast could result from the use of different materials and exposure times compared to the current study. The current findings partly contrasted those of Al-Zordk and Saker’s [55] who reported that coffee had a lower effect on the translucency parameter of thermocycled 5Y-TZP monolithic zirconia (Dental Direkt cubex^2^) compared to 3Y-TZP zirconia (Dental Direkt Bio Zx^2^). This discrepancy might arise from the utilization of distinct protocols and solutions.

Chlorhexidine mouthwash is usually recommended for a usage period of 7 to 14 days [56, 57]. However, its prolonged use for 28 to 42 days is associated with increased tooth staining, as reported earlier [58]. The current study simulated the clinical scenario of chlorhexidine use through 7 days of short-term exposure. The exact mechanism behind chlorhexidine-induced tooth discoloration remains unclear. However, it is hypothesized that the chlorhexidine molecules disintegrate within the oral cavity, forming parachloranilin, which could potentially induce protein denaturation and metal sulfides formation, leading to staining of teeth and restorations [59, 60].

The pH of the solution in which the acrylic resin is immersed has been reported as a significant factor influencing discoloration [61]. Additionally, mouthwashes with higher alcohol content induce more discoloration in bioceramic materials due to higher alcohol absorption and solubility [62–64]. Listerine contains around 30% alcohol, which could explain the substantial discoloration observed in the present and in some previous studies [37].

The present study assessed the color-difference by using a formula based on CIELAB, incorporating lightness, chroma, and hue weighting functions, along with an interactive term addressing chroma and hue differences to enhance the accuracy of assessment for blue color as well as a scaling factor for CIELAB a* scale to enhance the performance for gray colors. Nowadays the CIEDE2000 color difference formula has emerged and is recommended for its enhanced applicability and reliability in dentistry. Moreover, it offers improved adjustments for determining color differences by addressing non-uniformities in the CIELAB formula [65].

Among the limitations of the current study was its *in-vitro* nature, which allowed staining on both sides of the specimens, unlike clinical conditions. This factor also precluded the consideration of oral hygiene practices, like tooth brushing, which can affect restoration color stability *in vivo* [66]. Furthermore, in the current study, the CIELAB color difference formula was used to measure color differences, instead of the more recent CIEDE2000 color difference formula. Future studies are recommended to assess the impact of mouthwashes on the color stability and translucency of different types of monolithic zirconia in oral conditions using the advanced CIEDE2000 color difference formula.

## 5. Conclusions

Within the limitations of the present study and with respect to the evaluated materials, it can be concluded that high-translucency monolithic zirconia is more susceptible to discoloration when exposed to both chlorhexidine and Listerine mouthwashes than the other two monolithic zirconia systems (ML and LT). Meanwhile, multilayered monolithic zirconia is the most color stable material. Nevertheless, color changes in all groups were below the clinically acceptable threshold (ΔE< 3.7) Moreover, multilayered zirconia exhibits the greatest changes in translucency when exposed to chlorhexidine and Listerine mouthwashes compared to the other two monolithic zirconia systems (HT and LT).

## Supporting information

S1 Table Raw data obtained from all study groups. (XLSX)
